# Association between COX-2 rs2745557 polymorphism and prostate cancer risk: a systematic review and meta-analysis

**DOI:** 10.1186/1471-2172-13-14

**Published:** 2012-03-21

**Authors:** Hongtuan Zhang, Yong Xu, Zhihong Zhang, Ranlu Liu, Baojie Ma

**Affiliations:** 1 Department of Urology, Second Hospital of Tianjin Medical University, Tianjin Institute of Urology, Tianjin, China; 2 Department of Urology, Second Hospital of Tianjin Medical University, 23 Pingjiang Road, Hexi District, Tianjin 300211, China

## Abstract

**Background:**

Evidence is accumulating that chronic inflammation may have an important role in prostate cancer (PCa). The COX-2 polymorphism rs2745557 (+202 C/T) has been extensively investigated as a potential risk factor for PCa, but the results have thus far been inconclusive. This meta-analysis was performed to derive a more precise estimation of the association.

**Methods:**

A comprehensive search was conducted to identify all case-control studies of COX-2 rs2745557 polymorphism and PCa risk. We used odds ratios (ORs) to assess the strength of the association, and 95% confidence intervals (CIs) give a sense of the precision of the estimate. Statistical analyses were performed by Review Manage, version 5.0 and Stata 10.0.

**Results:**

A total of 8 available studies were considered in the present meta-analysis, with 11356 patients and 11641 controls for rs2745557. When all groups were pooled, there was no evidence that rs2745557 had significant association with PCa under co-dominant, recessive, over-dominant, and allelic models. However, our analysis suggested that rs2745557 was associated with a lower PCa risk under dominant model in overall population (OR = 0.85, 95%CI = 0.74-0.97, P = 0.02). When stratifying for race, there was a significant association between rs2745557 polymorphism and lower PCa risk in dominant model comparison in the subgroup of Caucasians (OR = 0.86, 95%CI = 0.75-0.99, P = 0.04), but not in co-dominant, recessive, over-dominant and allelic comparisons.

**Conclusion:**

Based on our meta-analysis, COX-2 rs2745557 was associated with a lower PCa risk under dominant model in Caucasians.

## Background

PCa is one of the most frequently diagnosed malignancies and a common cause of cancer mortality in men in the Western hemisphere [1,2]. Identifying risk factors for PCa is critically important to develop potential interventions and to expand our understanding of the biology of this disease. Despite the fact that the complex etiology of PCa remains obscure, various risk factors play an important role in PCa development such as advanced age, environmental variations, culture changes, and genetic variations. A strong association exists between states of chronic inflammation and cancer, and it is believed that mediators of inflammation may be responsible for this phenomenon [3]. Chronic inflammation may lead to tumorigenesis by damaging DNA through radical oxygen and nitrogen species, enhancing cell proliferation, and stimulating angiogenesis [4]. Some single nucleotide polymorphisms in specific cytokine genes have been proved to influence the expression and/or activity of encoding proteins probably making thereby the host predispose to certain cancer [5-7], so rs2745557 polymorphism of COX-2 that involved in the inflammatory pathway might impact susceptibility to PCa.

COX, also known as prostaglandin-endoperoxide synthase (PTGS), catalyzes the rate-limiting step in the formation of inflammatory prostaglandins. COX is an integral membrane bifunctional enzyme, which metabolizes arachidonic acids to many biologically active eicosanoids. COX-2 gene located on chromosome 1q25.2-q25.3 is a candidate gene for PCa susceptibility [8]. COX-2 is an inducible enzyme that converts arachidonic acid to prostaglandins, which play a role in cell proliferation and are potent mediators of inflammation. A meta-analysis suggested that aspirin use was associated a trend of decreased PCa risk [9]. The data suggested that COX-2 is overexpressed in PCa tissue compared to benign tissue from the same patient in several studies [10-14]. Some previous studies suggested that COX-2 may influence carcinogenesis by inhibiting apoptosis [15], inducing angiogenesis [16] and by chronic activation of immune responses [17].

Several polymorphisms in the COX-2 gene have been described, such as rs5277, rs689466, rs2206593, rs689470, and rs2745557. rs2745557 polymorphism in intron 1 has been brought to our attention. The functional impact of rs2745557, an intronic variant, on COX-2 activity is not yet known. Several studies were conducted to investigate the associations of COX-2 rs2745557 with PCa susceptibility [18-24]. However, molecular epidemiological studies have yielded contradictory results concerning potential roles of rs2745557 polymorphism in PCa. Individual studies might have been underpowered to detect the overall effects. Some studies are limited by their sample size and subsequently suffer from too low power to detect effects that may exist. Given the amount of accumulated data, we deemed it important to perform a quantitative synthesis of the evidence. Therefore, we performed this meta-analysis study to determine whether COX-2 rs2745557 was associated with PCa risk.

## Methods

### Literature search

We searched the articles using the terms "COX-2" or "PTGS2", "prostate", "carcinoma" or "cancer" or "tumor", and "polymorphism" or "variation" in PubMed, Cochrane Library and Embase electronic databases, and all eligible studies were published before November 15, 2011. We evaluated all associated publications to retrieve the most eligible literatures. The reference lists of reviews and retrieved articles were hand searched at the same time. We did not include abstracts or unpublished reports. When overlapping data of the same patient population were included in more than one publication, only the most recent or complete study was used in this meta-analysis. Articles were limited to English language papers.

### Inclusion and exclusion criteria

The following inclusion criteria were used to select literatures for the meta-analysis: (1) information on the evaluation of COX-2 rs2745557 polymorphism and PCa susceptibility; (2) case-control studies; and (3) sufficient genotype data were presented to calculate the OR with 95% CI. Major reasons for exclusion of studies were: (1) no controls; (2) reviews and duplication of the previous publication; and (3) no usable data reported.

### Data extraction

All data were extracted independently by two investigators according to the prespecified selection criteria. Disagreement was resolved by discussion. The following data were extracted: the name of the first author, publication year, ethnicity of the population, available genotype, number of prostate cancer cases and controls studied and results of studies. Different descents were categorized as Caucasian, Asian, and African American. For case-control studies, data were extracted separately for each group whenever possible.

### Statistical analysis

The strength of the association between COX-2 rs2745557 polymorphism and PCa risk was measured by ORs, whereas a sense of the precision of the estimate was given by 95% CIs. The significance of the summary OR was determined with a Z-test. We first examined rs2745557 genotypes using co-dominant model (homogeneous co-dominant model: TT vs CC, heterogeneous co-dominant model: TC vs CC), recessive (TT vs TC + CC), over-dominant (TC vs TT + CC) and dominant (TT + TC vs CC) genetic models. Then, the relationship between the allele and susceptibility to PCa was examined (allelic model). Stratified analyses were also performed by ethnicities. A chi-square-based Q-statistic test and an I^2^-test test were both performed to evaluate the between-study heterogeneity of the studies. In our study, two models of meta-analysis were applied for dichotomous outcomes: the fixed-effects model and the random-effects model. The fixed-effects model assumes that studies are sampled from populations with the same effect size, making an adjustment to the study weights according to the in-study variance. The random-effects model assumes that studies are taken from populations with varying effect sizes, calculating the study weights both from in-study and between-study variances, considering the extent of variation, or heterogeneity. A P-value ≥0.10 for the Q-test indicated lack of heterogeneity among the studies, and so the summary OR estimate of each study was calculated by the fixed-effects model [25]. Otherwise, the random-effects model (DerSimonian and Laird method) was used [26]. I^2 ^statistic can be used to quantify heterogeneity irrespective of the number of studies.

The significance of the pooled OR was determined by the Z-test and P < 0.05 was considered as statistically significant. To explore the reasons of heterogeneity, subgroup analyses were performed by ethnicity. The one-way sensitivity analyses were performed to assess the stability of the results, namely, a single study in the meta-analysis was deleted each time to reflect the influence of the individual data set to the pooled OR. To investigate whether publication bias might affect the validity of the estimates, funnel plot were constructed. An asymmetric plot suggests a possible publication bias. Funnel plot asymmetry was assessed by the method of Egger's linear regression test, a linear regression approach to measure funnel plot asymmetry on the natural logarithm scale of OR. The significance of the intercept was determined by the t-test suggested by Egger (P < 0.05 was considered representative of statistically significant publication bias). All statistical tests were performed with Review Manage, version 5.0 and Stata 10.0 using two-sided P-values.

## Results

### Eligible studies

According to the inclusion criteria defined above, we identified 8 independent studies in 7 eligible reports [18-24], including 11356 cases and 11641 controls. All the included 8 eligible reports were written in English. 8 independent studies consisted of 1 Asian, 1 African American and 6 Caucasian populations. 2 studies included in the subgroup analysis of Caucasians also contained a relevant proportion of subjects was not Caucasian [20,21]. Main characteristics for all eligible studies were listed in Table 1.

**Table 1:** Main characteristics of studies included in this meta-analysis

First author	Year	Country	Cases	Controls	Ethnicity	rs2745557 cases				rs2745557 controls			
						
						CC (%)	TC (%)	TC + TT (%)	TT (%)	CC (%)	TC (%)	TC + TT (%)	TT (%)
Wu et al.	2011	China	218	436	Asian	165(75.7)	49(22.5)	53(24.3)	4(1.8)	320(73.4)	107(24.5)	116(26.6)	9(2.1)
Amirian et al.	2011	USA	535	533	Caucasian	372(69.5)		163(30.5)		353(66.2)		180(33.8)	
Salinas et al.	2010	USA	335	396	Caucasian	225(67.2)		110(32.8)		251(63.4)		145(36.7)	
Fradet et al.	2009	USA	466	478	Caucasian (83%), African American	337(72.3)		129(27.7)		301(63.0)		177(37.0)	
Dossus et al.	2009	USA Europe	7941	8527	Caucasian, African-American, Latino, native Hawaiian, Asian	5614(70.7)	2098(26.4)	2327(29.3)	229(28.8)	5954(69.9)	2338(27.4)	2573(30.2)	235(2.8)
Cheng et al.	2007	USA	89	89	African American	69(77.5)	19(21.3)	20(22.5)	1(1.1)	56(63.0)	30(33.7)	33(37.1)	3(3.4)
Cheng et al.	2007	USA	417	417	Caucasian	295(70.7)	107(25.7)	122(29.3)	15(36.0)	262(62.8)	142(34.1)	155(37.2)	13(3.1)
Shahedi et al.	2006	Sweden	1355	765	Caucasian	945(69.7)	376(27.7)	410(30.3)	34(2.5)	545(71.2)	205(26.8)	220(28.8)	15(2.0)

### Meta-analyses

**Figure 1:**
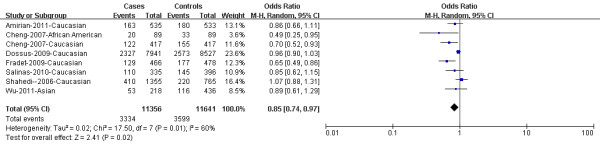
**Cox-2 rs2745557 was associated with a lower PCa risk under dominant model in overall population (TT + TC versus CC)**. The squares and horizontal lines correspond to the study specific OR and 95% CI. The area of the squares reflects the weight (inverse of the variance). The diamond represents the summary OR and 95% CI.

**Figure 2:**
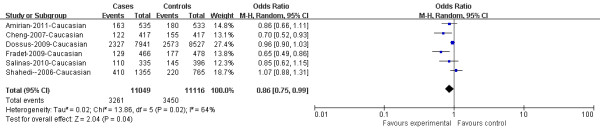
**Cox-2 rs2745557 was associated with a lower PCa risk under dominant model in Caucasians (TT + TC versus CC)**. The squares and horizontal lines correspond to the study specific OR and 95% CI. The area of the squares reflects the weight (inverse of the variance). The diamond represents the summary OR and 95% CI.

In overall population, there was significant heterogeneity in COX-2 rs2745557 for dominant model comparison, heterogeneous co-dominant model, over-dominant model and allelic model comparison, except for the homogeneous co-dominant model and recessive model comparisons. After subgroup analyses by ethnicity, the heterogeneity was effectively removed under allelic model in Caucasians. The data suggested that rs2745557 was associated with a lower PCa risk under dominant model in overall population (OR = 0.85, 95%CI = 0.74-0.97, P = 0.02; Figure 1). However, we did not detect the association between rs2745557 polymorphism and PCa risk in overall population when examining the contrast of TT versus CC, TC versus CC, TT versus TC + CC, TC versus TT + CC, and T allele versus C allele (OR = 1.04, 95% CI = 0.88-1.23, P =0.66; OR = 0.88, 95% CI = 0.74-1.04, P = 0.14; OR = 1.06, 95% CI = 0.89-1.25, P = 0.51; OR = 0.88, 95% CI = 0.75-1.04, P = 0.13; and OR = 0.92, 95% CI = 0.80-1.06, P = 0.26, respectively). Similarly, in subgroup analyses stratified by ethnicity, the remarkable association with lower PCa risk was detected in dominant model comparison in Caucasian population (OR = 0.86, 95%CI = 0.75-0.99, P = 0.04; Figure 2). No noteworthy associations were observed under co-dominant, recessive, over-dominant, and allelic models in Caucasians. The detailed data were presented in Table 2.

**Table 2:** Meta-Analysis of COX-2 rs2745557 Polymorphisms and Prostate Cancer

Genetic model (No. of studies)	Sample size		Analysis model	Test of association		P value for Egger's test	Test for heterogeneity	
					
	Case	Control		OR (95% CI)	P		P	I^2^
Total (8)								
TT vs CC (5)	7371	7412	F	1.04 (0.88-1.23)	0.66	0.510	0.74	0%
TC vs CC (5)	9737	9959	R	0.88 (0.74-1.04)	0.14	0.275	0.05	58%
TT vs TC + CC (5)	10020	10234	F	1.06 (0.89-1.25)	0.51	0.627	0.81	0%
TT + TC vs CC (8)	11356	11641	R	0.85 (0.74-0.97)	0.02	0.062	0.01	60%
TC vs TT + CC (5)	10020	10234	R	0.88 (0.75-1.04)	0.13	0.276	0.06	56%
T vs C (5)	20040	20468	R	0.92 (0.80-1.06)	0.26	0.284	0.07	55%
Caucasian (6)								
TT vs CC (3)	7132	7024	F	1.05 (0.89-1.25)	0.56		0.77	0%
TC vs CC (3)	9435	9446	R	0.91 (0.76-1.10)	0.33		0.04	68%
TT vs TC + CC (3)	9713	9709	F	1.07 (0.90-1.27)	0.43		0.80	0%
TT+TC vs CC (6)	11049	11116	R	0.86 (0.75-0.99)	0.04		0.02	64%
TC vs TT + CC (3)	9713	9709	R	0.91 (0.76-1.09)	0.31		0.04	68%
T vs C (3)	19426	19418	F	0.97 (0.92-1.03)	0.32		0.11	55%

*OR *odds ratio, *CI *confidence interval, *vs *versus, *R*, random effect model, *F*, fixed effect model

### Sensitivity analysis

In order to compare the difference and evaluate the sensitivity of the meta-analyses, we conducted one-way sensitivity analysis to evaluate the stability of the meta-analysis. The statistical significance of the results was not altered when any single study was omitted (data not shown), confirming the stability of the results. Hence, results of the sensitivity analysis suggest that the data in this meta-analysis are relatively stable and credible.

### Publication bias

Begg's funnel plot and Egger's test were performed to assess the publication bias. The shape of funnel plots did not reveal any evidence of obvious asymmetry in all comparison models, and the Egger's test was used to provide statistical evidence of funnel plot symmetry. The results did not show any evidence of publication bias. The detailed data were present in Table 2.

## Discussion

We consider the COX-2 gene highly interesting in the search for susceptibility genes for PCa. Previous study results suggested that single nucleotide polymorphisms (SNPs) are the most common sources of human genetic variation, and they may contribute to an individual's susceptibility to cancer [27]. In the recent years, interest in the genetic susceptibility to cancers has led to a growing attention to the study of polymorphisms of genes involved in tumourigenesis. Since the identification of COX-2 rs2745557 polymorphism, growing number of studies suggested that COX-2 rs2745557 polymorphism plays an important role in the development of PCa. Epidemiological studies of the rs2745557 polymorphism in COX-2, if large and unbiased, can provide insight into the in vivo relationship between the gene and PCa risk. However, these studies have appeared in the literature either supporting or negating the significant association. Some reviewed studies are limited by their sample size and subsequently suffer from too low power to detect effects that may exist. But the pool ORs generated from much larger population can increase the statistical power. Combining data from many studies has the advantage of reducing random error [28].

In order to provide the comprehensive and reliable conclusion, we performed the present meta-analysis of 8 independent case-control studies [18-24], including 11356 patients and 11641 controls. According to the study design, 3 studies were conducted in a population-based design [20,22,24], and 5 in a hospital-based design [18,19,21,23]. Some studies reported insufficient information about recruitment methodology and study participant characteristics, particularly for controls. The control populations were not uniform. Healthy populations as well as non-cancer patients were included. Some individuals in the control group are likely to develop cancer in subsequent years though they had no clinical symptoms at the time of investigation. Our results indicated that the rs2745557 was associated with a lower PCa risk under dominant model in overall population. Nevertheless, considering that rs2745557 polymorphism may play different roles in PCa susceptibility among different ethnic subgroups and the frequencies of rs2745557 polymorphism might be different among different ethnic groups which might contribute to the possible presence of heterogeneity between the studies, we further conducted subgroup analysis by ethnicity in current meta-analysis. In the stratified analysis by ethnicity, our results suggested that COX-2 rs2745557 polymorphism was associated with a lower PCa risk among subjects of Caucasians. We found that COX-2 rs2745557 polymorphism was associated with a trend of decreased PCa risk under dominant model and allelic model in African Americans, however significant relation was absent in Asians. There may be many factors influencing the result, such as differences in populations, selection factors and so on. The rs2745557 was associated with a lower PCa risk under dominant model. However, the significant association is completely lost under homogeneous co-dominant model or allelic model. So we can speculate that rs2745557 may associate with a lower PCa risk under over-dominant model. However, the significant association was absent. The reason for this phenomenon may be caused by a lack of sufficient genotype data in several studies. Considering the limited studies and population numbers of African Americans and Asians included in the meta-analysis, this may increase the risk of false negative findings, any conclusions at overall population level should be interpreted with caution. Therefore, we are not sure whether there is a significant association between the COX-2 polymorphism and decreased PCa risk in the whole population due to low statistical power.

Heterogeneity is a potential problem when interpreting the results of the present meta-analysis. In overall analysis, significant between-study heterogeneity existed in dominant model, heterogeneous co-dominant model, over-dominant model and allelic model comparisons. After subgroup analyses by ethnicity, the heterogeneity was removed under allelic model in Caucasians. In this meta-analysis, high levels of heterogeneity were observed in some comparisons. There are some factors that could have contributed toward the high heterogeneity. First, there is likely to be considerable genetic heterogeneity between the samples that were drawn from geographically diverse populations. It is known that genotype distributions differ across populations, and genotype-phenotype associations may also depend on population stratification. Second, definition of control group is different in different studies, the definition differences of the controls could have contributed to the high heterogeneity observed in our meta-analysis. Third, we attempted to determine if the high heterogeneity might also be explained by other variables such as stages of PCa, smoking status, and environmental factors included in the different studies, but are unable to provide a reliable answer to this question because we did not have access to individual level data for these variables.

Some limitations of this meta-analysis should be acknowledged. First, because only published and English articles were included in the meta-analysis, publication and potential English language biases may have occurred, even though it was not found by making use of statistical test. Second, the result should be cautiously interpreted because controls were not uniformly defined. Non-differential misclassification bias was possible because these studies may have included controls that had different risks for developing PCa. Third, only one study analyzed Asian population and another one African American population in this study. So it is quite important to have more studies and sample of Asians, Africans, and African Americans in the future so that more precise conclusion about the associations between rs2745557 polymorphism and PCa risk could be achieved. Fourth, our results were based on unadjusted estimates, while a more precise analysis should be conducted adjusted by other factors like smoking, drinking status and environmental factors. In addition, our analysis did not consider the possibility of gene-gene or SNP-SNP interactions or the possibility of linkage disequilibrium between polymorphisms. Further investigations of the haplotypic effect of a gene and the study of multiple polymorphisms in different genes are needed.

## Conclusions

In conclusion, our meta-analysis suggested that there was an association between rs2745557 polymorphism and lower PCa risk in Caucasians. Due to limitations showed above in this analysis, it is critical that larger and well-designed multicenter studies are needed to confirm our results.

## Abbreviations

PCa: Prostate cancer; PTGS: Prostaglandin-endoperoxide synthase; OR: odds ratio; CI: Confidence interval; vs: Versus; R: Random effect model; F: Fixed effect model.

## Competing interests

The authors declare that they have no competing interests.

## Authors' contributions

ZH carried out the publication search, participated in data analysis and drafted the manuscript. XY carried out publication search, and revised the manuscript. ZZ and LR participated in the publication search and helped to draft the manuscript. MB participated performed the statistical analysis. All authors read and approved the final manuscript.
